# Severe Psychomotor Delay in a Severe Presentation of Cat-Eye Syndrome

**DOI:** 10.1155/2015/943905

**Published:** 2015-01-14

**Authors:** Guillaume Jedraszak, Aline Receveur, Joris Andrieux, Michèle Mathieu-Dramard, Henri Copin, Gilles Morin

**Affiliations:** ^1^Unité de Génétique Médicale et Oncogénétique, Centre Hospitalier Universitaire Amiens-Picardie, 80054 Amiens Cedex, France; ^2^Laboratoire de Cytogénétique et Biologie de la Reproduction, CECOS de Picardie, Centre Hospitalier Universitaire Amiens-Picardie, 80054 Amiens Cedex, France; ^3^Laboratoire de Génétique Médicale, Hôpital Jeanne de Flandre, Centre Hospitalier Régional Universitaire de Lille, 59037 Lille Cedex, France

## Abstract

Cat-eye syndrome is a rare genetic syndrome of chromosomal origin. Individuals with cat-eye syndrome are characterized by the presence of preauricular pits and/or tags, anal atresia, and iris coloboma. Many reported cases also presented with variable congenital anomalies and intellectual disability. Most patients diagnosed with CES carry a small supernumerary bisatellited marker chromosome, resulting in partial tetrasomy of 22p-22q11.21. There are two types of small supernumerary marker chromosome, depending on the breakpoint site. In a very small proportion of cases, other cytogenetic anomalies are reportedly associated with the cat-eye syndrome phenotype. Here, we report a patient with cat-eye syndrome caused by a type 1 small supernumerary marker chromosome. The phenotype was atypical and included a severe developmental delay. The use of array comparative genomic hybridization ruled out the involvement of another chromosomal imbalance in the neurological phenotype. In the literature, only a few patients with cat-eye syndrome present with a severe developmental delay, and all of the latter carried an atypical partial trisomy 22 or an uncharacterized small supernumerary marker chromosome. Hence, this is the first report of a severe neurological phenotype in cat-eye syndrome with a typical type 1 small supernumerary marker chromosome. Our observation clearly complicates prognostic assessment, particularly when cat-eye syndrome is diagnosed prenatally.

## 1. Introduction

Cat-eye syndrome (CES), also referred to as Schmid-Fraccaro syndrome (OMIM 115470), is a rare genetic disease with an estimated prevalence of between 1 in 50,000 and 1 in 150,000 individuals [1]. Individuals with CES are characterized by three main clinical features: preauricular pits and/or tags, anal atresia, and iris coloboma. However, many reported cases also feature congenital kidney abnormalities, congenital cardiac defects, intellectual disability, and growth delay. It has been observed that most patients diagnosed with CES carry a small supernumerary bisatellited marker chromosome (sSMC), which results in partial tetrasomy of 22p-22q11.21. There are two types of sSMC, depending on the breakpoint site: type 1, the most frequent, involves the cat-eye syndrome critical region (CESCR) alone, whereas type 2, more rarely reported, involves both the CESCR and the DiGeorge syndrome critical region [2]. Other exceptional cytogenetic anomalies, such as partial trisomy of chromosome 22 [3] and intrachromosomal triplication of 22q11.21 region [4], are also reportedly associated with the CES phenotype.

Here, we report a patient who presented with typical features of CES: imperforate anus, severe preauricular and auricular anomalies, and cardiac malformation. Progression of the syndrome was marked by an uncommon, severe psychomotor delay. Cytogenetic analyses (including karyotyping, fluorescence* in situ* hybridization (FISH), and array comparative genomic hybridization (CGH)) revealed a typical type 1  sSMC involving the 22p-22q11.21 region and ruled out other chromosomal imbalances.

## 2. Case Presentation

**Figure 1:**
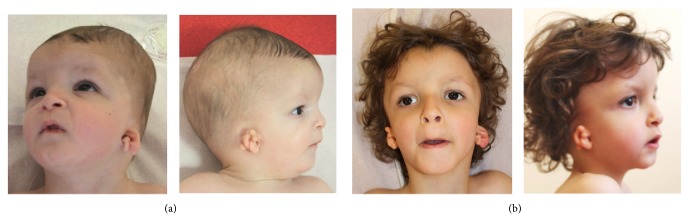
Craniofacial dysmorphism. Pictures of the patient at the age of 6 months (top panel) and 3 and a half (bottom panel). The facial dysmorphism consisted in hypertelorism, downslanting palpebral fissures, a thin upper lip, retrognathism, an irregularly shaped skull, and severe malformation of the external ears.

The patient was the second child born to healthy, unrelated parents with no family history of malformation or intellectual disability. The pregnancy featured an elevated risk score at the second-trimester trisomy 21 screening test (1 out of 198) and the development of intrauterine growth restriction during the third trimester. The parents did not wish amniocentesis to be performed. The patient was born after 38 weeks of gestation, with a birth weight of 2800 g (10th percentile) and a head circumference of 35 cm (50–75th percentile). A clinical examination revealed an imperforate anus, facial dysmorphism (Figure 1), general hypotonia, and bilateral malformation of the external ears (present as several tags, in combination with atresia of the external auditory canal) (Figure 1). Hearing tests evidenced a 70 dB bilateral conductive hearing loss. A CT scan showed bilateral hypoplasia of the tympanic cavity and right-side hypoplasia of the middle ear. Due to poor weight gain during the first weeks of life, the patient underwent gastrostomy. The subsequent course was marked by a severe global developmental delay: the child began sitting at the age of 2 years and 9 months and was unable to walk at the age of 3 and a half. His language was also severely impaired, with the absence of distinctive words until the age of 3 years and a half. Brain MRI showed thickening of the upper two-thirds of the pituitary stalk but no other malformation, suggesting the presence of an ectopic posterior pituitary gland in addition to the normally situated posterior pituitary gland. The results of hormone assays (for IgF1, GH, ACTH, FSH, LH, TSH, FT3, FT4, and cortisol) were normal. Cryptorchidism and right-side Duane syndrome were also observed. At the age of 3 and a half, the patient weighed 10.4 kg (<3rd percentile), was 97 cm tall (25th–50th percentile), and had a head circumference of 49 cm (25th–50th percentile).

**Figure 2:**
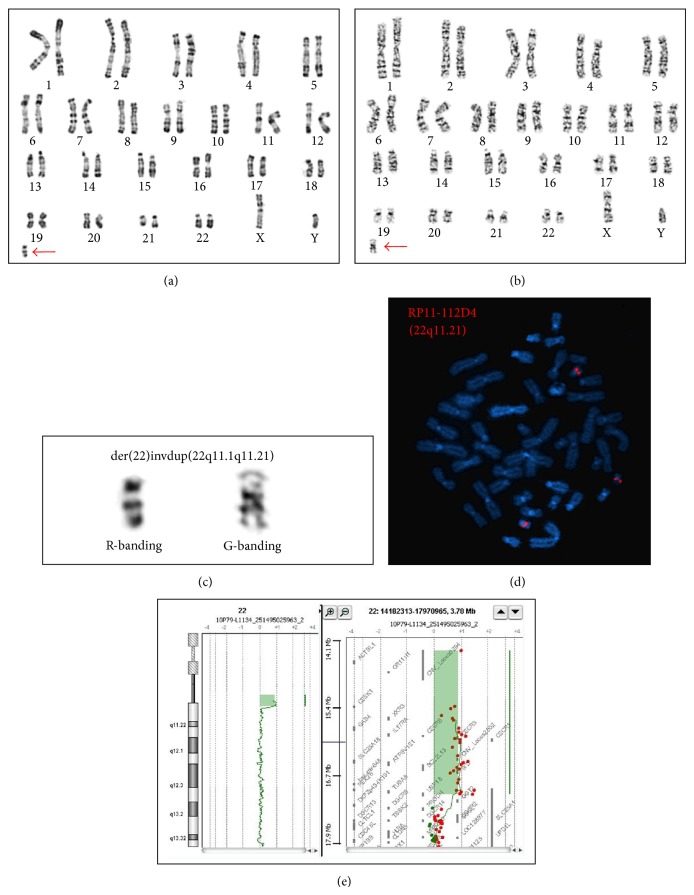
Cytogenetic testing. RHG-banding (a) and GTG-banding (b) karyotypes show the sSMC (red arrow). A zoomed image on the sSMC confirmed that the latter is dicentric and bisatellited (c). FISH analysis with an RP11-112D4 (22q11.21) probe shows two normal signals and one doubled signal, confirming the involvement of the CESCR (d).

Karyotyping of a peripheral blood sample revealed an additional dicentric sSMC (Figures 2(a), 2(b), and 2(c)). The results of FISH analyses using probes RP11-112D4 (22q11.21—cat-eye syndrome critical region) and TBX1 (DiGeorge syndrome critical region) (Figure 2(d)) and oligonucleotide-based array-CGH using a 44K array (AgilentTM, Agilent Technologies, Santa Clara, CA, USA) showed a typical type 1 CES chromosome (Figure 2(e)), with the following results: 47,XY,+idic(22)(q11.21)[19].ish22q11.21(RP11-112D4)x4[15].arr22q11.1q11.21(16,053,473-18,641,468)x4(hg19). The parents' karyotypes were normal, confirming the* de novo* occurrence of the sSMC.

## 3. Discussion

To the best of our knowledge, this is the first report of a very severe neurological phenotype in CES caused by an isolated type 1  sSMC (according to McTaggart et al.'s classification [2]).

The three main characteristic clinical symptoms identifying cat-eye syndrome are preauricular anomalies, anorectal malformations, and coloboma of the iris. Other recurrent observed features include variable congenital kidney abnormalities, congenital cardiac defects, and mild to severe growth delays. Symptoms and findings associated with CES are also extremely variable in range and severity among the affected patients.

This phenotypic variability in CES has been extensively studied [1, 5]. Our patient manifested only two of the three typical characteristics: an anal malformation and ear anomalies. Missing of one of the three main clinical sign is not so uncommon in CES patients: only 41% of them presented with the three main characteristic features. Iris coloboma is the most frequently missing typical feature, as 50% of patients with CES do not present with this eye anomaly [1]. Furthermore, the patient presented with other features commonly found in CES, such as cryptorchidism (24% of cases), impaired ocular motility (25 to 76% of cases), and facial dysmorphism.

Intellectual disability or psychomotor delay (ID/PD) can also be considered as a common feature in CES, since it is present in 32% of cases. However, the neurologic impairment is rarely prominent. Of the 50 patients carrying invdup(22) and with a detailed neurologic phenotype reviewed in [1], only 17 presented with mild-to-moderate ID/PD and none presented with severe ID/PD. Only a few patients diagnosed with CES have been reported as suffering from a severe developmental delay. These patients did not carry the common type 1 sSMC but carried other chromosomal anomalies rarely reported to be associated with the CES phenotype: partial trisomy 22q (more often associated with severe ID/DD [6, 7]) or a type 2  sSMC [8]. It is noteworthy that none of these patients have been assessed with array-CGH; hence, the presence of an additional, small, associated chromosomal imbalance (which may have been involved in the severe neurological phenotype) cannot be ruled out.

We reported the first case of severe developmental delay in a patient with CES caused by a typical type 1 sSMC. Absence of mosaicism for the sSMC could explain a part of the severity of the phenotype of our patient, even if previous reported studies did not show any impact of the mosaicism rate on the severity of the phenotype of CES patients [1]. Array-CGH ruled out the involvement of another chromosomal imbalance in the neurological phenotype. However, we cannot exclude the involvement of other genetics (point mutation in one of the genes comprised in the sSMC or in another gene responsible for developmental delay, uniparental disomy, etc.) or nongenetic factors in the severity of the phenotype of our patient.

In conclusion, this observation clearly complicates prognostic assessment when CES is diagnosed prenatally.
